# Poster Session II - A260 ASSESSING THE RELATIONSHIP BETWEEN FCP, CRP, AND THE HARVEY BRADSHAW INDEX IN PREGNANT PATIENTS WITH CROHN’S DISEASE

**DOI:** 10.1093/jcag/gwaf042.260

**Published:** 2026-02-13

**Authors:** K Kecskemeti, H Nabavian, S Eisen, V Srikanth, K O’ Connor, V W Huang

**Affiliations:** Faculty of Medicine, University of Toronto, Toronto, ON, Canada; Faculty of Medicine, University of Toronto, Toronto, ON, Canada; Faculty of Medicine, University of Toronto, Toronto, ON, Canada; Faculty of Medicine, University of Toronto, Toronto, ON, Canada; Faculty of Medicine, University of Toronto, Toronto, ON, Canada; University of Toronto, Toronto, ON, Canada

## Abstract

**Background:**

Crohn’s Disease (CD) predominantly affects women during their reproductive years, making disease monitoring during pregnancy essential. However, pregnancy-related hormonal changes and fetal development influence symptoms of CD and disease activity.

**Aims:**

This research study aims to assess the correlation between fecal calprotectin (FCP), C-reactive protein (CRP) and the Harvey-Bradshaw Index (HBI) for pregnant patients with Crohn’s disease. We also aim to investigate FCP thresholds for clinically active disease during pregnancy.

**Methods:**

We identified all pregnant patients with Crohn’s disease who were seen at the Mount Sinai Hospital Pregnancy IBD clinic from January 2017 to December 2022. We analyzed demographic characteristics including age and parity. Biochemical markers FCP and CRP were recorded at each trimester. We collected clinical HBI scores which were recorded during clinic visits at each trimester and gathered from the outpatient EMR. We defined active clinical disease if a patient had a HBI ≥ 5 or a clinician-documented impression of active disease. Spearman’s rho tests were performed to analyze the correlation between the HBI scores, FCP and CRP for each trimester. We completed a bivariate assessment with Chi-Squared tests to determine FCP cut-offs for patients with active and inactive clinical disease activity during pregnancy.

**Results:**

A total of 155 pregnant patients with CD were included in the study. The mean maternal age at conception was 33.2 ± 4.1 years. First-time pregnancies accounted for 96 cases (64%). Table 1 documents the Spearman’s correlation coefficients between HBI scores and FCP values. All three trimesters had negative non-significant correlations. Table 2 demonstrates weakly non-significant correlation between HBI scores and FCP in trimester 1 and 2. In the third trimester there was a positive significant correlation between CRP and HBI scores.

To evaluate FCP thresholds for clinical activity during pregnancy, various values were investigated. There was a statistically significant difference between clinically active vs inactive disease observed in all trimesters with an FCP threshold of 300 µg/g (Table 3). A threshold value of 250 µg/g and 150 µg/g were not statistically significant in the first trimester.

**Conclusions:**

For pregnant patients with CD, the Harvey Bradshaw Index is mostly discordant with objective disease markers of disease activity such as CRP and FCP during pregnancy. These results highlight the need for objective biochemical monitoring of disease activity in during pregnancy. A statistically significant difference was observed in all trimesters using an FCP level of 300 µg/g. Future research should be conducted to determine the impact of these thresholds on maternal and fetal pregnancy outcomes in patients with CD.

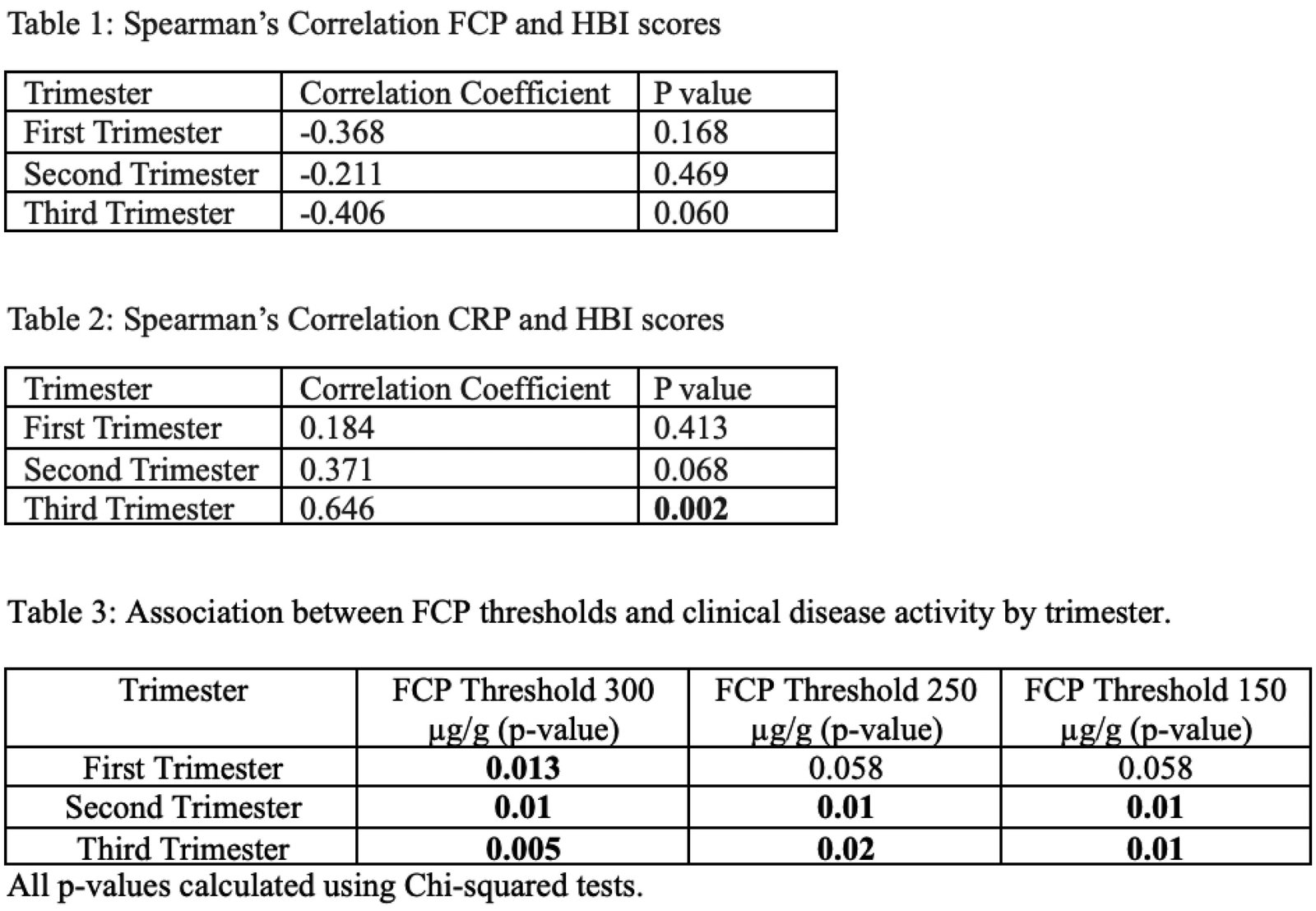

**Funding Agencies:**

Mount Sinai Hospital Resident Research Scholarship

